# OSU53 Rescues Human OB-6 Osteoblastic Cells from Dexamethasone through Activating AMPK Signaling

**DOI:** 10.1371/journal.pone.0162694

**Published:** 2016-09-15

**Authors:** Dawei Xu, Wei Zhao, Xinhui Zhu, Jianbo Fan, Shengyu Cui, Yuyu Sun, Xiang Chen, Wei Liu, Zhi-ming Cui

**Affiliations:** The Department of Orthopaedics, The Second Affiliated Hospital of Nantong University, Nantong, 226001, China; Suzhou University, CHINA

## Abstract

Excessive dexamethasone (Dex) application causes osteoblast cell death, which could lead to osteoporosis or osteonecrosis. AMP-activated protein kinase (AMPK) activation is shown to protect osteoblasts/osteoblastic cells from Dex. In this report, we tested the potential effect of OSU53, a novel AMPK activator, in Dex-treated osteoblastic cells. We show that OSU53 activated AMPK signaling in human OB-6 osteoblastic cells. Further, Dex-induced osteoblastic OB-6 cell death and apoptosis were largely attenuated with pre-treatment with OSU53. OSU53 was more efficient than other known AMPK activators (A-769662 and Compound 13) in protecting OB-6 cells against Dex. AMPK activation is required for OSU53-induced actions in OB-6 cells. AMPKα shRNA knockdown or dominant-negative mutation (dn-AMPKα T172A) almost completely blocked OSU53-induced AMPK activation and OB-6 cell protection against Dex. Further studies showed that OSU53 increased NADPH (nicotinamide adenine dinucleotide phosphate) activity and alleviated Dex-induced oxidative stress in OB-6 cells. Such effects by OSU53 were again almost abolished with AMPKα shRNA or dn-AMPKα in OB-6 cells. Together, these results demonstrate that OSU53 protects osteoblastic cells from Dex possibly via activating AMPK-dependent signaling.

## 1. Introduction

Dexamethasone (Dex) and other glucocorticoids (GCs) are common anti-inflammatory and immuno-suppressive medicines [1]. Yet, prolonged and/or excessive GC application could lead to osteoporosis [2,3] or even osteonecrosis [4]. Dex is known to induce cytotoxic effects to osteoblasts, which contributes to subsequent bone damages [5,6]. Dex was also added to cultured osteoblasts/osteoblastic cells to imitate GC-induced bone damages [5,7,8,9,10]. Groups including ours [10,11] are focusing on the pathological mechanisms of GC-induced osteoblast damages, and on developing possible intervention strategies [9,10,11,12,13,14].

AMP-activated protein kinase (AMPK) is the master regulator of energy and metabolism in eukaryotic cells [15,16,17]. It has been recently proposed that AMPK and it-regulated signalings are also vital for cell survival [18]. Activation of AMPK could attenuate oxidant stress via activating nicotinamide adenine dinucleotide phosphate (NADPH) and reducing ATP consumption [19]. Meanwhile, activated AMPK could also provoke cytoprotective autophagy to recycle the cellular components [20,21]. On the other hand, AMPK inhibition, depletion or mutation would exacerbate cell damages by various stresses [20,21].

Recent research has focused on the potential activity of AMPK in osteoblasts/osteoblastic cells. Guo et al., showed that Compound 13 (C13), a novel AMPK activator [22], protected osteoblasts from Dex via activating of AMPK signaling [8]. Zhu et al., found that A-769662, another known AMPK activator, inhibited hydrogen dioxide (H_2_O_2_)-induced osteoblastic cell death [23]. Reversely, She et al, showed that genetic or pharmacological inhibition of AMPK exacerbated H_2_O_2_-induced osteoblastic cell damages [24]. Therefore, AMPK activation exerts a cytoprotective role in osteoblasts/osteoblastic cells. Recently, Chen’s group has developed a novel AMPK activator, name OSU53 [25]. In the present study, we show that OSU53 also protects osteoblastic cells from Dex.

## 2. Materials and Methods

### 2.1. Chemicals and reagents

OSU53 was synthesized according to a published procedure [25] by Ming-de Biotech (Soochow, China). For all experiments, OSU53 was dissolved in DMSO, and added to cells at a final DMSO concentration of 0.1%. Dex was purchased from Sigma Aldrich (Shanghai, China). The antibodies in this study were purchased from Cellular Signaling Tech (Shanghai, China). Other known AMPK activators A769662 and Compound 13 were provided as gifts from Dr. Guo’s lab [8].

### 2.2. Cell culture

The human osteoblastic cell line OB-6 [5] was obtained from the Cell Bank of Shanghai Institute of Biological Science (Shanghai, China). The OB-6 cells were cultured as previously described [5]. The cell culture reagents were purchased from Gibco (Shanghai, China).

### 2.3. Western blot assay

As described in our previous studies [10,11], cell lysates (30 μg total proteins per lane) were electro-transferred via 10–12% SDS-PAGE gel, following by transfer onto PVDF membranes. The blots were then incubated with designated primary and secondary antibodies. The antigen-antibody binding was detected via enhanced chemiluminescence (ECL) reagents. ImageJ software was applied to quantify band’s total gray.

### 2.4. Cell death detection

Following the designated treatment, OB-6 osteoblastic cell death was tested by trypan blue dye assay, which stained the cytoplasm of dead cells. Cell death ratio (%) = the number of trypan blue stained cells/the number of total cells (×100%) [11].

### 2.5. Cell survival assay

Following treatment of cells, the viability was measured via routine 3-[4,5-dimethylthylthiazol-2-yl]-2,5 diphenyltetrazolium bromide (MTT) assay described in our previous studies [10,11].

### 2.6. Apoptosis assay by enzyme-linked immunosorbent assay (ELISA)

We applied the Histone-DNA Apoptosis ELISA Detection Kit (Roche, Palo Alto, CA) to quantify cell apoptosis. The detailed protocol was described in our previous studies [10,11].

### 2.7. Stable knockdown AMPKα by shRNA

The two lentiviral shRNAs (GV248-puromycin vector) against human AMPKα were designed, synthesized and verified by Genepharm Co. (Shanghai, China). The OB-6 cells were seeded onto 6-well plates with 50% of confluence. The lentiviral shRNA (10 μL/mL) was added directly to the OB-6 cells for 24 hours. Afterwards, cells were cultured in puromycin (1 μg/mL)-containing medium (with FBS). After 10–14 days, the resistant colonies were formed. AMPKα expression in the stable cells was tested by Western blot assay. The scramble lentiviral shRNA (Santa Cruz) was added to the control OB-6 cells.

### 2.8. AMPK dominant-negative mutation

The dominant-negative mutant of AMPKα (dn-AMPKα, T172A, flag-tagged) construct was a gift from Dr. Lu’s group at Nanjing Medical University (see [26]). The construct or the empty vector was transfected to OB-6 cells via Lipofectamine 2000 protocol [26]. Stable cells expressing dn-AMPKα were selected via neomycin (1 μg/mL, Sigma). Expression of dn-AMPKα was verified via Western blot assay. Control OB-6 cells was transfected with empty vector (“Vector”, pSuper-neo).

### 2.9. NADPH activity assay

Assay of NADPH activity was described in previous literatures [24,27]. NADPH activity in the treatment cells was normalized to that of untreated control cells.

### 2.10. Reactive oxygen species (ROS) intensity assay

ROS intensity was measured via a DCFH-DA fluorescent dye (Invitrogen, Shanghai, China). After treatment, cells were incubated with 1 μM of DCFH-DA (Invitrogen) for 30 min. Cells were then washed and analyzed for fluorescence via a Fluorescence/Multi-Detection Microplate Reader (Synergy 2, BioTek, Winooski, VT). The ROS intensity OD in the treatment group was normalized to that of control group.

### 2.11. Statistics

The data presented were mean ± standard deviation (SD). Statistical differences were analyzed by one-way ANOVA followed by multiple comparisons performed with post hoc Bonferroni test (SPSS) [10,11]. Values of *p* < 0.05 were considered statistically significant.

## 3. Results

### 3.1. OSU53 activates AMPK in human osteoblastic cells

**Fig 1:**
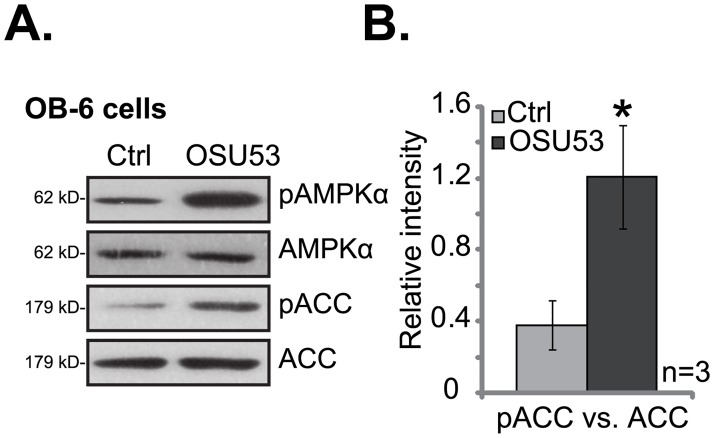
OSU53 activates AMPK in human osteoblastic cells. Human osteoblastic OB-6 cells were treated with or without OSU53 (10 μM) for two hours, expression of listed proteins was tested by Western blots (A). p-ACC level (vs. regular ACC) was quantified (B). “Ctrl” stands for untreated control group (Same for all figures). Experiments in this figure were repeated three times, and similar results were obtained. *p<0.05 vs. “Ctrl” group (B).

OSU53 is a newly-developed AMPK activator [25], we first tested its effect on AMPK activation in cultured osteoblastic cells. The OB-6 cells [5,28] were well-established human osteoblastic cells, these cells were treated with OSU53. Western blot results in Fig 1A showed that OSU53 at 10 μM [25] significantly increased phosphorylations (p-) of AMPKα (Thr-172) and its major downstream acetyl-CoA carboxylase (ACC, Ser-79) in OB-6 cells, indicating a significant AMPK activation. The concentration of OSU53 (10 μM) was chosen based on previous study [25]. On the other hand, expression of regular AMPK and ACC was not affected by OSU53 treatment (Fig 1A). p-ACC level (vs. regular ACC) was quantified in this study to reflect AMPK activation intensity (Fig 1B). p-ACC intensity increased over three times following OSU53 treatment in OB-6 cells (Fig 1B). Similarly in hFOB1.19 osteoblastic cells, treatment with OSU53 induced significant AMPK and ACC phosphorylations (Data not shown). Together, these results show that OSU53 activates AMPK in human osteoblastic cells.

### 3.2. OSU53 protects human osteoblastic cells from Dex

**Fig 2:**
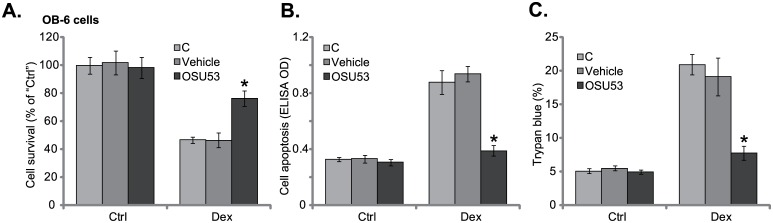
OSU53 protects human osteoblastic cells from Dex. Human osteoblastic OB-6 cells were pretreated for 1 hour with OSU53 (10 μM), followed by Dex (1 μM) stimulation for 24 hours, cell viability (MTT assay, A), cell apoptosis (Histone DNA ELISA assay, B) and cell death (trypan blue assay, C) were tested. Experiments in this figure were repeated three times, and similar results were obtained. “C” stands for no intervention. *p<0.05 vs. cells with Dex only treatment.

It has been shown that AMPK activation could protect osteoblasts/osteoblastic cells from Dex [8,23,24], we then wanted to know if OSU53 could exert similar functions. In line with our previous findings [10,11], treatment OB-6 cells with Dex (1 μM) for 24 hours induced significant viability reduction (MTT OD decrease, Fig 2A), cell apoptosis (Histone DNA ELISA OD increase, Fig 2B) and cell death (Trypan blue ratio increase, Fig 2C). Remarkably, pre-treatment with OSU53 (10 μM) dramatically attenuated Dex-exerted OB-6 cell death and apoptosis (Fig 2A–2C). Similar results were also obtained in hFOB1.19 osteoblastic cells, where OSU53 pre-treatment significantly alleviated Dex-mediated cell damages (Data not shown). As expected, the vehicle control (0.1% DMSO) showed no effect on Dex in the osteoblastic cells (Fig 2A–2C). Together, these results show that OSU53 efficiently protects human osteoblastic cells from Dex.

### 3.3. OSU53 is more efficient than other known AMPK activators in protecting OB-6 cells against Dex

**Fig 3:**
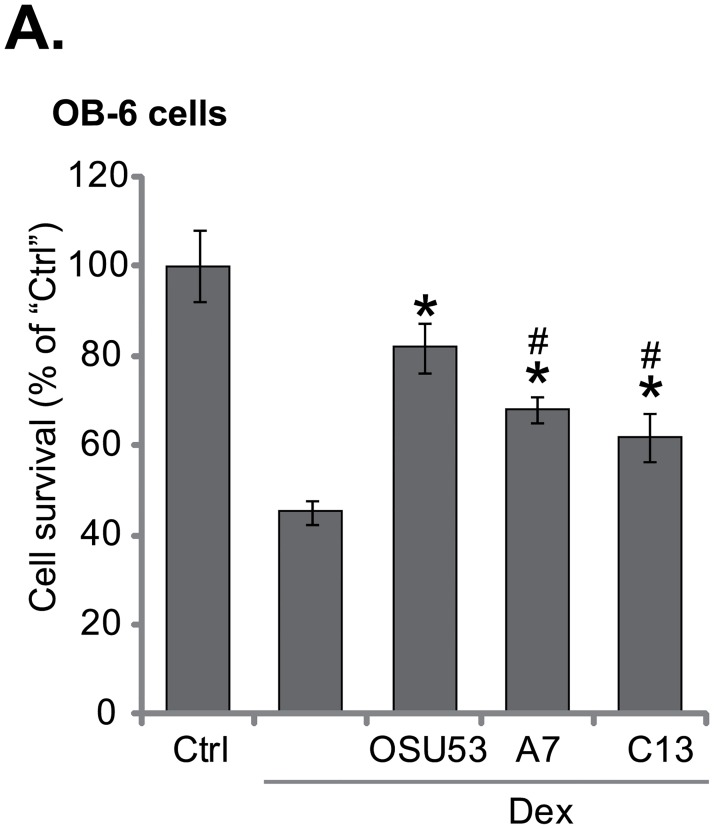
OSU53 is more efficient than other known AMPK activators in protecting OB-6 cells against Dex. OB-6 cells were pretreated with OSU53 (10 μM), A-769662 (“A7”, 10 μM) or Compound 13 (“C13”, 10 μM) for 1 hour, followed by Dex (1 μM) stimulation for 24 hours, cell viability (MTT assay) was shown (A). Experiments in this figure were repeated three times, and similar results were obtained. *p<0.05 vs. “Dex” only treatment. ^#^ *p*<0.05 vs. OSU53 treatment group.

We compared OSU53 with other known AMPK activators in Dex-treated osteoblastic cells. Two other AMPK activators, A-769662 (“A7”, 10 μM) [23] and Compound 13 (“C13”, 10 μM) [8], also attenuated Dex-induced OB16 cell death (Fig 3A). Significantly, same concentration of OSU53 (10 μM) was more potent than the two (“A7” and “C13”) in protecting OB16 cells (Fig 3A). These results indicate that OSU53 is better than other known AMPK activators in protecting OB-6 cells.

### 3.4. AMPK activation is required for OSU53-induced OB-6 cell protection against Dex

**Fig 4:**
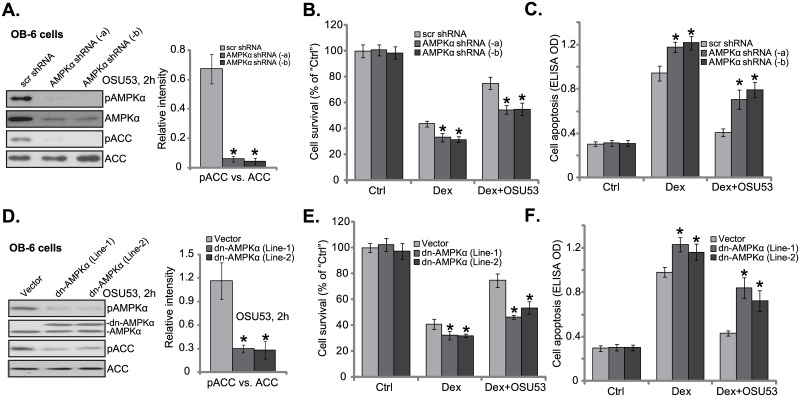
AMPK activation is required for OSU53-induced osteoblastic cell protection against Dex. Stable OB-6 cells, expressing AMPKα shRNA (“-a, or–b”, with non-overlapping sequences) or the scramble control shRNA (“scr shRNA”), were treated with OSU53 (10 μM) for 2 hours, expression of listed proteins was tested by Western blots (A); Above cells were also treated with Dex (1 μM) or plus OSU53 (10 μM, 1 hour pretreatment) for 24 hours, cell survival (MTT assay, B) and apoptosis (Histone DNA ELISA assay, C) were tested. Stable OB-6 cells, expressing dn-AMPKα (“Line-1 or Line-2”) or the empty vector (pSuper-neo, “Vector”), were treated with OSU53 (10 μM) for 2 hours, expression of listed proteins was tested by Western blots (D); Cells were also treated with Dex (1 μM) or plus OSU53 (10 μM, 1 hour pretreatment) for 24 hours, cell survival (E) and apoptosis (F) were tested. p-ACC level (vs. regular ACC) was quantified (A and D). Experiments in this figure were repeated three times, and similar results were obtained. “Ctrl” stands for untreated control group. *p<0.05 vs. “scr shRNA” or “Vector” cells.

Above results have shown that OSU53 activated AMPK in human osteoblastic cells. Further, this novel AMPK activator protected osteoblastic cells from Dex. We next wanted to know if activation of AMPK is required for OSU53’s activity. To block AMPK activation, we utilized genetic strategies. Two non-overlapping lentiviral AMPKα shRNAs (“-a” or “-b”) were introduced to OB-6 cells, and stable cells expressing these shRNAs were established. As shown in Fig 4A, the AMPKα shRNA (“-a” or “-b”) both dramatically downregulated AMPKα expression in OB-6 cells. As a result, OSU53-indued AMPK activation (or AMPK/ACC phosphorylation) was almost blocked (Fig 4A). Thus, AMPKα subunit is required for OSU53-induced AMPK activation. Significantly, OSU53-induced cytoprotection against Dex was almost nullified in AMPKα-silenced OB-6 cells (Fig 4B and 4C). In another words, OSU53 was in-effective in AMPKα knockdown cells (Fig 4B and 4C).

Next, a dominant-negative AMPKα (“dn-AMPKα”, T172A) [26] was introduced to OB-6 cells. Western blot results in Fig 4D confirmed dn-AMPKα (flag-tagged) expression in OB-6 cells. We were able to establish two OB-6 cell lines expressing dn-AMPKα (“Line-1 and Line-2”). As demonstrated, OSU53-indued AMPK activation was largely inhibited in the dn-AMPKα-expressing OB-6 cells (Fig 4D). As a result, OSU53-mediated cytoprotection against Dex was also dramatically attenuated (Fig 4E and 4F). Notably, Dex-induced OB-6 cell death (Fig 4B and 4E) and apoptosis (Fig 4C and 4F) were exacerbated with AMPKα knockdown or mutation, indicating that basal AMPK activation is important for osteoblastic cell survival against Dex. Collectively, we suggest that AMPK activation is required for OSU53-induced osteoblastic cell protection against Dex.

### 3.5. OSU53 activates AMPK to alleviate Dex-mediated oxidative stress in OB-6 cells

**Fig 5:**
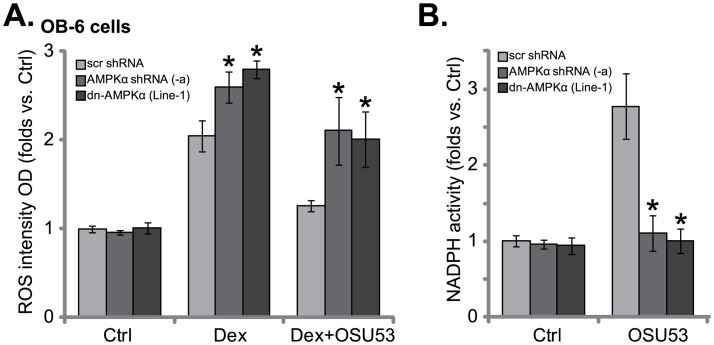
OSU53 activates AMPK to alleviate Dex-mediated oxidative stress in osteoblastic cells. Stable OB-6 cells expressing dominant-negative AMPKα (“dn-AMPKα”, T172A, “Line-1”), AMPKα shRNA (“-a”), or the scramble control shRNA (“scr shRNA”) were treated with Dex (1 μM) or plus OSU53 (10 μM, 1 hour pretreatment), ROS intensity (DCFH-DA fluorescent OD, 6 hours, A) was tested; Relative NADPH activity in above cells was also shown (4 hours, B). Experiments in this figure were repeated three times, and similar results were obtained. *p<0.05 vs. “scr shRNA” cells.

Recent studies have shown that Dex induces oxidative stress in osteoblasts/osteoblastic cells [8]. In the present study, a significant ROS production was also noticed in Dex-treated OB-6 cells (Fig 5A). Significantly, pre-treatment with OSU53 in OB-6 cells largely attenuated Dex-mediated ROS production (Fig 5A). OSU53’s mediated anti-oxidant activity was dependent on AMPK. AMPKα shRNA knockdown or dominant-negative mutation almost abolished OSU53’s anti-oxidant ability (Fig 5A). Since AMPK exerts an anti-oxidant function via activating NADPH [8,19,24,29]. We next analyzed NADPH activity in OSU53-treated OB-6 cells. As demonstrated, OSU53 increased NADPH activity in OB-6 cells (Fig 5B) AMPKα shRNA knockdown or mutation almost reversed OSU53-induced NADPH activation (Fig 5B). These results show that OSU53 activates AMPK-NADPH signaling to possibly alleviate Dex-mediated oxidative stress in osteoblastic cells, and eventually protects cells from Dex.

## 4. Discussions

Osteoblasts are mesenchymal progenitor cells-derived cells, which are essential for maintain bone growth [10,30,31]. Yet, osteoblasts are also the main target cells of Dex in the bone [10,30,31]. Osteoblast cell depletion and increased osteoblast cell death/apoptosis were observed in Dex-taking patients’ bones [2,3,4]. Previous *in vitro* studies [7,9,10,11] have demonstrated that Dex can induce direct damages to cultured osteoblasts/osteoblastic cells. In this report, we show that OSU53, a novel AMPK activator, activated AMPK signaling and attenuated Dex-induced osteoblastic cell death and apoptosis. AMPK activation is required for OSU53-induced functions in osteoblastic cells. On the other hand, AMPKα shRNA knockdown or dominant-negative mutation in OB-6 cells almost completely blocked OSU53-induced osteoblastic cytoprotection against Dex.

Dex kills osteoblasts/osteoblastic cells partly via provoking oxidative stress [8,32]. ROS scavengers, on the other hand, are shown to protect osteoblasts/osteoblastic cells from Dex [8]. Recent publications have implied that activated-AMPK may function as a potent anti-oxidant signaling, and protects cells from a number of stress conditions [8,19,24,33]. In this regard, AMPK will phosphorylate and inhibit its major downstream ACC to decrease NADPH consumption [19]. Meanwhile, AMPK could also promote NADPH synthesis via fatty-acid oxidation [19]. Consequently, NADPH activity is increased following AMPK activation [19]. In this report, our results demonstrated that OSU53 activated AMPK-NADPH signaling and suppressed Dex-induced ROS production. This should be accountable, at least in part, for its cytoprotection effects in OB-6 osteoblastic cells. Significantly, inhibition of AMPK by AMPKα shRNA or dn-AMPKα almost abolished OSU53-mediated anti-oxidant and cytoprotective activities in OB-6 cells. Therefore, AMPK activation is required for OSU53-mediated anti-oxidant activity.

## 5. Conclusions

We conclude that OSU53 protects osteoblastic cells from Dex possibly via activating AMPK-dependent anti-oxidant signalings. Although many AMPK specific activators have been developed and are being tested in preclinical studies, there are few specific AMPK activators entering clinical trials for various diseases [22]. Therefore, it will be interesting to test the possible effect of this novel AMPK activator OSU53 against GC-induced osteoporosis and/or osteonecrosis *in vivo*.
